# Development of correlations between various engineering rockmass classification systems using railway tunnel data in Garhwal Himalaya, India

**DOI:** 10.1038/s41598-024-60289-y

**Published:** 2024-05-10

**Authors:** Md. Alquamar Azad, Taoufik Najeh, Autar K. Raina, Neelratan Singh, Abdullah Ansari, Mujahid Ali, Yewuhalashet Fissha, Yaser Gamil, S. K. Singh

**Affiliations:** 1 Department of Geology (Centre for Advanced Studies), University of Delhi, Delhi, 110007 India; 2 Operation and Maintenance, Operation, Maintenance and Acoustics, Department of Civil, Environmental and Natural Resources Engineering, Lulea University of Technology, Lulea, Sweden; 3 CSIR-Central Institute of Mining and Fuel Research (Ministry of Science & Technology, Govt. of India), Nagpur Research Center 17/C, Telangkhedi Area, Civil Lines, Nagpur, Maharashtra 440001 India; 4 Academy of Scientific and Innovative Research (AcSIR), CSIR-Human Resource Development Centre (CSIR-HRDC) Campus, Postal Staff College Area, Sector 19, Kamla Nehru Nagar, Ghaziabad, Uttar Pradesh 201 002 India; 5 Earthquake Monitoring Center, Sultan Qaboos University, Muscat, 123 Oman; 6 Department of Transport Systems, Traffic Engineering and Logistics, Silesian University of Technology, Krasińskiego 8 Street, Katowice, Poland; 7 Department of Geosciences, Geotechnology and Materials Engineering for Resources, Graduate School of International Resource Sciences, Akita University, Akita, 010-8502 Japan; 8 Department of Civil Engineering, School of Engineering, Monash University Malaysia, Jalan Lagoon Selatan, 47500 Bandar Sunway, Selangor Malaysia; 9 Department of Civil Engineering, Inha University, Incheon, 22212 South Korea

**Keywords:** Rockmass classification, Metamorphic rock, Tunnelling, Garhwal Himalaya, Engineering geology, Engineering, Geology

## Abstract

Engineering rockmass classifications are an integral part of design, support and excavation procedures of tunnels, mines, and other underground structures. These classifications are directly linked to ground reaction and support requirements. Various classification systems are in practice and are still evolving. As different classifications serve different purposes, it is imperative to establish inter-correlatability between them. The rating systems and engineering judgements influence the assignment of ratings owing to cognition. To understand the existing correlation between different classification systems, the existing correlations were evaluated with the help of data of 34 locations along a 618-m-long railway tunnel in the Garhwal Himalaya of India and new correlations were developed between different rock classifications. The analysis indicates that certain correlations, such as RMR-Q, RMR-RMi, RMi-Q, and RSR-Q, are comparable to the previously established relationships, while others, such as RSR-RMR, RCR-Qn, and GSI-RMR, show weak correlations. These deviations in published correlations may be due to individual parameters of estimation or measurement errors. Further, incompatible classification systems exhibited low correlations. Thus, the study highlights a need to revisit existing correlations, particularly for rockmass conditions that are extremely complex, and the predictability of existing correlations exhibit high variations. In addition to augmenting the existing database, new correlations for metamorphic rocks in the Himalayan region have been developed and presented that can serve as a guide for future rock engineering projects in such formations and aid in developing appropriate excavation and rock support methodologies.

## Introduction

Any engineering design and analysis requires numeric information. Rockmass, that is the fundamental design variable in any excavation, however, presents verbal descriptions of many of its properties. This leads to classifications that enables conversion of the description of rocks into numbers, that in turn can be used for engineering analysis. Broadly, engineering rockmass classifications of empirical nature provide means to:

- Assign numerical values to rockmass properties, assign ratings, define the class of rock for engineering analysis and design,
- Define the strength of the rockmass while considering all field properties and joints observed for stability of the excavations in a particular class of rockmass, and
- Define the modulus, in situ strength of the rockmass, standup time and even coefficient and angle of internal friction
- Define the excavability and support in a particular type of rockmass, so that the rockmass withstands the stresses related to excavations of varied types including, mining, civil and defence constructions.

Rockmass classifications not only bridge the communication between civil engineers and geologists, but offer correlations for engineering practice and help to better the organization of knowledge of rockmass properties, also. This is perhaps the best explanation as to why, quantitative rockmass classification methods remain useful in rock engineering^ 1–3^. A comprehensive evaluation of a multitude of rockmass classification systems has been brought out by Palmström^3^, Singh and Goel^2^ and Sadeghi et al.^1^.

Many such classification systems have evolved over the past and only a few are popular. These classifications rely on few determinable laboratory or field scale numeric, and other properties that are descriptive in nature^4^. All such properties are then assigned ratings and the sum or product of such ratings are defined over a definite range to evaluate the rock class.

Some of the rockmass classifications that have received significant attention from engineering geologists and civil engineers are the Rockmass Rating or RMR^5–8^, the Rockmass Quality or Q, Rock Mass Index or RMi^9^ and Geological Strength Index or GSI^ 9, 10^. Other important classification systems include Rock Structure Rating, RSR^10,11^, Mining Rock Mass Rating, MRMR^12^, Modified Basic RMR, MBR^12^, Slope Rock Mass Rating, SRMR^13^, Rock Mass Number, Q_n_^14^, Rock Condition Rating, RCR^3^ and Rock Mass Strength, RMS^15^.

**Figure 1:**
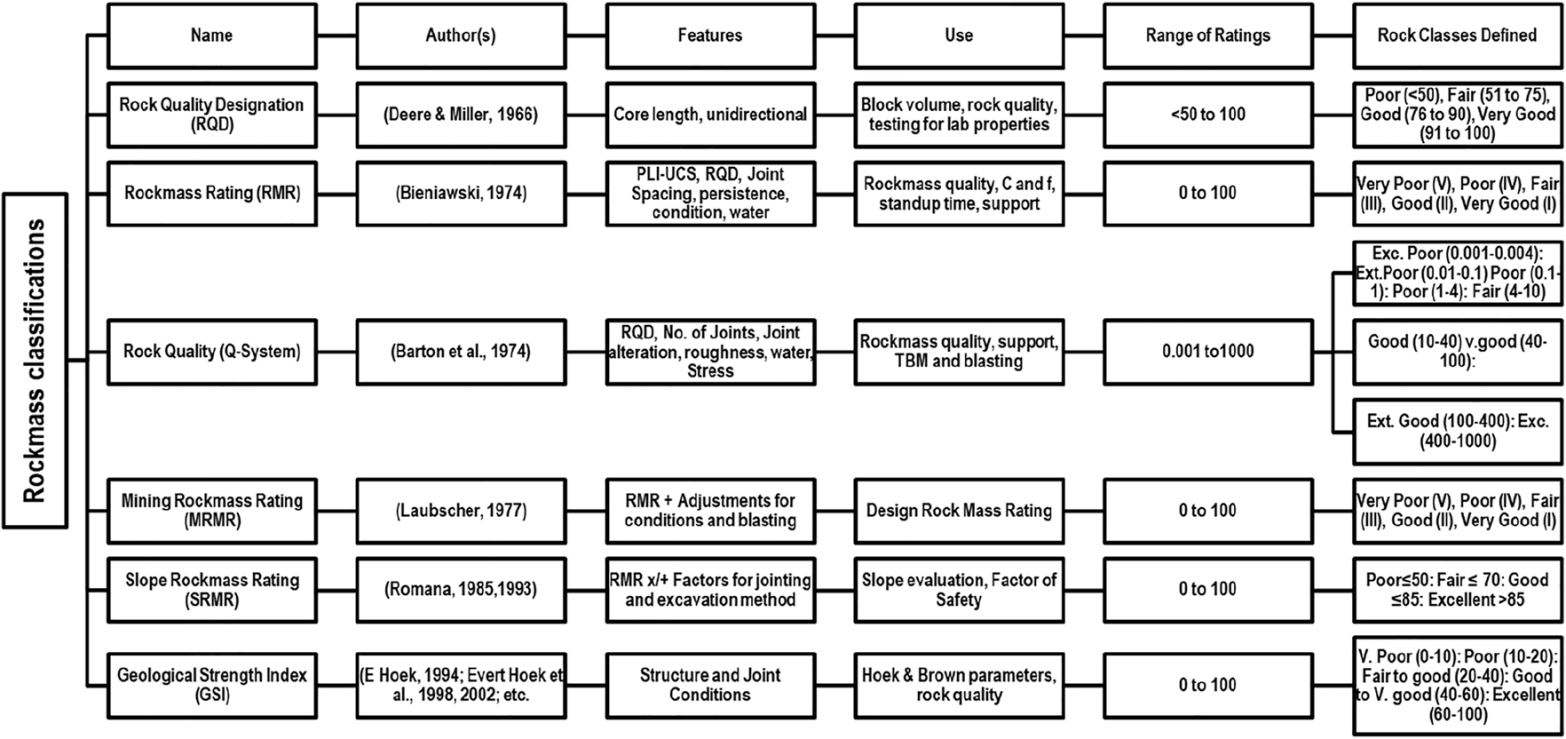
Some important and popular rockmass classification systems, their features, uses and range of classes (modified after Bhatawdekar et al.^6^*).*

It will not be out of place to mention that the literature on rockmass classifications and their uses is too exhaustive and practically difficult to summarize in a single publication. However, a summary of some of the popular rockmass classifications and their further adaptations are presented in Fig. 1, wherein, the features, use, range of ratings and rock classes defined by the respective authors, have been provided.

The classification systems employ different sets of variables of the rock or rockmass (around 10), ranging from in situ block size, joint conditions, and the intact strength of the rockmass etc., as compiled in Table 1.

**Table 1:** Variables considered in several classification systems for underground excavations, modified after Sadeghi et al.^1^.

Sl. No	Rockmass classification ►	RQD	RSR	RMR	RMR_B_	Q	Qn	MRMR	RMS	MBR	SRMR	SMR	GSI	RCR	RMi	Total^a^
1.	Variables used ▼	1	2	3	4	5	6	7	8	9	10	11	12	13	14	
2.	Block size	×	×	×	×	×	×	×	×	√	×	×	×	×	√	2
3.	Joint orientation	×	√	√	×	×	×	×	×	√	×	×	×	×	√	4
4.	No. of joint sets	×	×	×	×	√	√	×	√	×	×	×	×	√	√	5
5.	Joint length	×	×	×	×	×	×	×	×	×	×	×	×	×	√	1
6.	Joint spacing	√	√	√	√	√	√	√	√	√	√	√	√	√	√	**14**
7.	Joint strength	×	√	√	√	√	√	√	√	√	√	√	√	√	√	**13**
8.	Rock type	×	√	×	×	×	×	×	×	×	×	×	×	×	×	1
9.	State of stress	×	×	×	×	√	×	√	×	√	×	×	×	×	×	3
10.	Ground water condition	×	√	√	√	√	√	√	√	√	√	√	×	√	×	**11**
11.	Strength of intact rock	×	×	√	√	×	√	√	√	√	√	√	×	√	√	**10**
12.	Total No. of variables used	1	5	5	4	4	5	5	5	**7**	4	4	2	5	**7**	

*RQD* rock quality designation, *RSR* rock structure rating, *RMR* rock mass rating, *RMR* basic form of RMR, *Q* tunnelling quality index, *Qn* rock mass number, *MRMR* mining rock mass rating, *RMS* rock mass strength, *MBR* modified basic rock mass rating, *SRMR* simplified rock mass rating, *SMR* slope mass rating, *GSI* geological strength index, *RCR* rock condition rating, *RMi* is rock mass index.

^a^Total classifications using the variable.

Significant values are in bold.

From Table 1, it can be observed that most of the classifications are relying on joint spacing, joint strength, while many of these give significant importance to the ground water condition and strength of the intact rock. RMi and MBR classifications use seven variables while as RQD and GSI are relying on one to two variables. This probably points to the different approaches used by the developers of the classification or a general disagreement on role of such variables in the classifications. However, it is imperative that joint length or persistence and joint condition are uniformly agreed to be a common factor in such classifications.

The classifications, e.g., as mentioned in Fig. 1, are invariably used in mining, civil construction, and rock engineering projects for evaluation of the ground response of underground structures like underground mine workings, tunnels, defence installations, slope stabilization in open pit mines and road alignments and in defining the excavability of the rockmass. In addition, many rockmass parameters may be derived from such rockmass classification systems, e.g., rockmass stability^16,17^, rockmass strength^18^ shear strength of rockmass^19^, deformation modulus^20–23^, angle of internal friction^24^, ultimate bearing pressure^25^, etc.

Despite the umpteen applications of the rockmass classification systems e.g., Q-system^26^, RMR^7^, GSI^27^, SRMR^28^, SRC for tectonic conditions^29^, and combination of Q and RMR^30^, Coal RMR^31,32^ and their advantages documented, the classification have their disadvantages as listed in Table 2. There have been attempts to further enhance the classification systems using artificial neural networks and related methods^33,34^ in recent times. However, the efficacy of such methods needs to be verified and validated through significant number of applications. These may however, be specific to a particular formation or project and will need some gestation period for defining their universal applicability.

**Table 2:** Advantages and disadvantages of the prominent rockmass classification systems in practice.

Sl. no.	Classification name	Advantages	Disadvantages
1	RMR	Comprehensive assessment of rock mass behavior Relatively easy to apply in the field Widely accepted and used in geotechnical practice	Subjectivity in rating assignment Limited applicability to certain geological conditions Relies on empirical data and may not always reflect site-specific conditions Does not account for dynamic changes in rock masses
3	Q-system	Focuses on block size, its interactions with joint conditions, water conditions and stress It is relatively simple and straight forward to apply, making it accessible to a wide range of professionals involve in rock engineering and excavation projects	Subjective interpretation of parameters such as joint roughness and joint alteration can introduce variability in the classification results, affecting the reliability of predictions
2	GSI	GSI provides a quantitative measure of rock mass strength and behavior based on geological characteristics such as joint parameters, and weathering conditions Engineers and geologists can use the information by rock GSI to optimize the design of civil structures and support systems	GSI requires detailed geological mapping and characterization of rock mass properties, which can be time consuming and challenging in some field conditions
4	SRMR	SRMR accounts for site-specific conditions, enabling tailored assessments based on the unique characteristics of each rock slope	SRMR calculation can be complex and require expertise in rock mechanics and geotechnical engineering for accurate assessment
5	MRMR	Allows for customization based on specific project requirements and geological conditions, providing more relevant and reliable assessments for tunneling, mining	The accuracy of MRMR assessments depends on the availability and quality of geological and geotechnical data which may not always be readily accessible or comprehensive, particularly in field-based evaluation

Different authors have tried to correlate rockmass classifications with each other with a good degree of correlation possibly because all the variables entering in one classification may not be characterized properly or represented in other classifications and to work out the inter-dependability of such systems for ease of use at a particular site. A comprehensive list of such correlations is presented in Table 3.

**Table 3:** Correlation of different rockmass classifications system (various authors).

References	Equation	*R*^*2*^	rock type
Bieniawski^6^	1. *RMR* = *9 ln(Q)* + *44*	0.77	Variety of rock types
Rutledge and Preston^54^	2. *RMR* = *5.9 ln(Q)* + *43* 3. *RSR* = *0.77RMR* + *12.4* 4. *RSR* = *13.3ln(Q)* + *46.5*	0.81 0.81	Multiple rock types, siltstone, quartzite
Cameron-Clarke and Budavari^54^	5. *RMR* = *5 ln(Q)* + *60.8*	NA	Volcanics, diabase
Abad et al.^55^	6. *RMR* = *10.5 ln(Q)* + *41.8*	0.66	Not provided
Kaiser and Gale^56^	7. *RMR* = *8.7 ln(Q)* + *38*	0.55	
Bieniawski^7^	8. *RCR* = *0.77RMR* = *12.4*	NA	Variety of rock types
Al-Harthi^57^	9. *RMR* = *9 ln(Q)* + *49*	NA	Sedimentary rock
Choquet and Hadjigeorgiou^58^	10. *RMR* = *12.5 ln(Q)* + *55.2* 11. *RMR* = *12.11 ln(Q)* + *50.81* 12. *RMR* = *10 ln(Q)* + *39*	NA	From different sources and varied rock types
El-Naqa^59^	13. *RMR* = *7 ln(Q)* + *44*	NA	Marly limestone
Barton^60^	14. *RMR* = *15 ln(Q)* + *50*	NA	General equation, multiple rocks
Goel et al.^15^	15. *RCR* = *8 ln(Qn)* + *30*	0.92	Multiple case studies
Tuǧrul^46 ^	16. *RMR* = *7 ln(Q)* + *36* 17. *RSR* = *0.78RMR* + *17* 18. *RSR* = *6 ln(Q)* + *46*	NA	Limestone
Sari and Pasamehmetoglu	19. *RCR* = *1.7 ln(Qn)* + *51.5* 20. *RMR* = *3.7 ln(Q)* + *53.1*	0.65 0.86	Limestone
Kumar et al.^47^	21. *RMR* = *6.4 ln(Q)* + *49.6* 22. *RMR* = *5.4RMi* + *54.4* 23. *RMi* = *0.5Q*^*0.93*^ 24. *RMi* = *1.5Q*^*0.72*^ 25. *RCR* = *8 ln(Qn)* + *42.7*	0.72 0.77	Gneisses, quartz mica schist, amphibolites, schist, quartzite
Morales et al.^60^	26. *GSI* = *4.714* + *0.687RMR*	*0.94*	Sandstone, siltstone, marl, claystone
Cosar^49^	27. *RMR* = *2.8 ln(Q)* + *45.19* 28. *GSI* = *0.42RMR* + *23.07* 29. *GSI* = *1.61 ln(Q)* + *42.99*		Schists with recrystallized limestone intercalation
Osgoui and Unal^61^	*30. GSI* = *6e*^*0.05RMR*^	*NA*	Metasiltstone, clayey and silty sandstone, shale and phyllite
Hashemi et al.^48^	*31. RMR* = *5.37 ln(Q)* + *40.48* *32. RMR* + *7.5 lnRMi* + *36.8* *33. RMi* = *1.082Q*^*0.4945*^ *34. RCR* = *6 ln(Qn)* + *33.84* *35. GSI* = *0.692RMR*_*89*_ + *22.32* *36. GSI* = *0.917GSI*_*(Cai)*_ + *3.18*	*0.53* *0.48* *0.53* *0.35* *0.74* *0.81*	Mostly limestone
Laderian and Abaspoor^62^	*37. RMR* = *8.15 ln(Q)* + *44.88* *38. RMR* = *42.87Q*^*0.16*^	*0.859*	Limestone, conglomerate, schist, sandstone etc
Ranasooriya and Nikraz^63^	*39. RMR* = *6.3 ln(Q)* + *43*	*NA*	Data from other sources
Rafiee et al.^64^	*40. RMR* = *8.09 ln(Q)* + *43.08*	*NA*	Multiple tunnels
Irvani et al.^65^	*41. GSI* = *1.35RMR* − *16.4*	*NA*	Granite
Caicedo and Pérez^66^	*42. RMR* = *5.7 ln(Q)* + *43.65*	*0.82*	Not known
Singh and Tamrakar^67^	*43. GSI* = *0.73RMR* − *4.38*	*NA*	Slate, dolomite, phyllite, quartzite, limestone, metasandstone
Ali et al.^68^	*44. RMR* = *2.87 ln(Q)* + *48.71* *45. GSI* = *0.99RMR* − *4.9*	*0.20* *0.84*	Norite
Senra^69^	*46. RMR* = *6.55 ln(Q)* + *59.53*	*NA*	Amphibolite, schist
Sayeed and Khanna^70^	*47. RMR* = *4.52 ln(Q)* + *43.635*	*0.74*	Various rock types
Zhang et al.^71^	*48. GSI* = *1.21RMR *−* 18.61*	*NA*	Different rock types

*Q *Q-system, *RSR* rock structure rating, *RMR*_*B*_ basic form of rock mass rating, *Qn* rock mass number, *RMR* rock mass rating, *MRMR* modified rockmass rating classification, *MBR* modified basic rockmass rating classification, *RMS* rock mass strength, *SRMR* simplified rockmass rating, *GSI *geological strength index, *GSI* (*Cai*) third method of geological strength index^20^, *RMi *rock mass index, *RCR* rock condition rating, *RSR *rock structure rating, *SMR *slope mass rating, *NA* not available or provided by the author.

As is evident from Table 3, as many as 28 authors proposed at least 49 correlations between various classification systems. The correlations mostly relate to prediction of RMR from the respective systems and could be for establishing their method as RMR is a comprehensive system and is widely used.

However, many of the correlations between different Rock mass classifications thus developed were not consistent (Table 3) or do not present high-order correlations. Such errors can arise from degree of exposure of rock, joints and other features, assessment of properties by individuals on a small scale and averaging of extreme data. In addition, the conversion of qualitative information into numbers is subjective and varies from person to person.

Rock types and the presence of significant variations in the independent variables along with human errors could also be the possible reason for the deviations^35^. It was with this intent that^14^ redefined RCR based on RMR and Qn from Q to obtain better correlations with GSI.

Accordingly, the objective of this work was to define the best possible correlations between various engineering rockmass classifications and to find their inter-correlatability particularly in metamorphic rocks in Himalayan conditions, this not only provides for new methods of inter-correlations of different Rock mass classifications but, also, augments the database and correlations for specific rock types. Also, the key differences in variables that can impact the strength of the intercorrelations have been explored. It will assist in choosing the most effective classification system for tunnel constructions in Himalayas.

Several relationships based on data obtained from a tunnelling project, including RMR-RCR, RMi-Q, RMR-RSR, Q-RSR, RMR-Q, RCR-RSR, RCR-Q, RMi-RSR that presents excellent inter-correlation and others that do not show significant correlation have thus been presented and evaluated in this study.

## Study area

**Figure 2:**
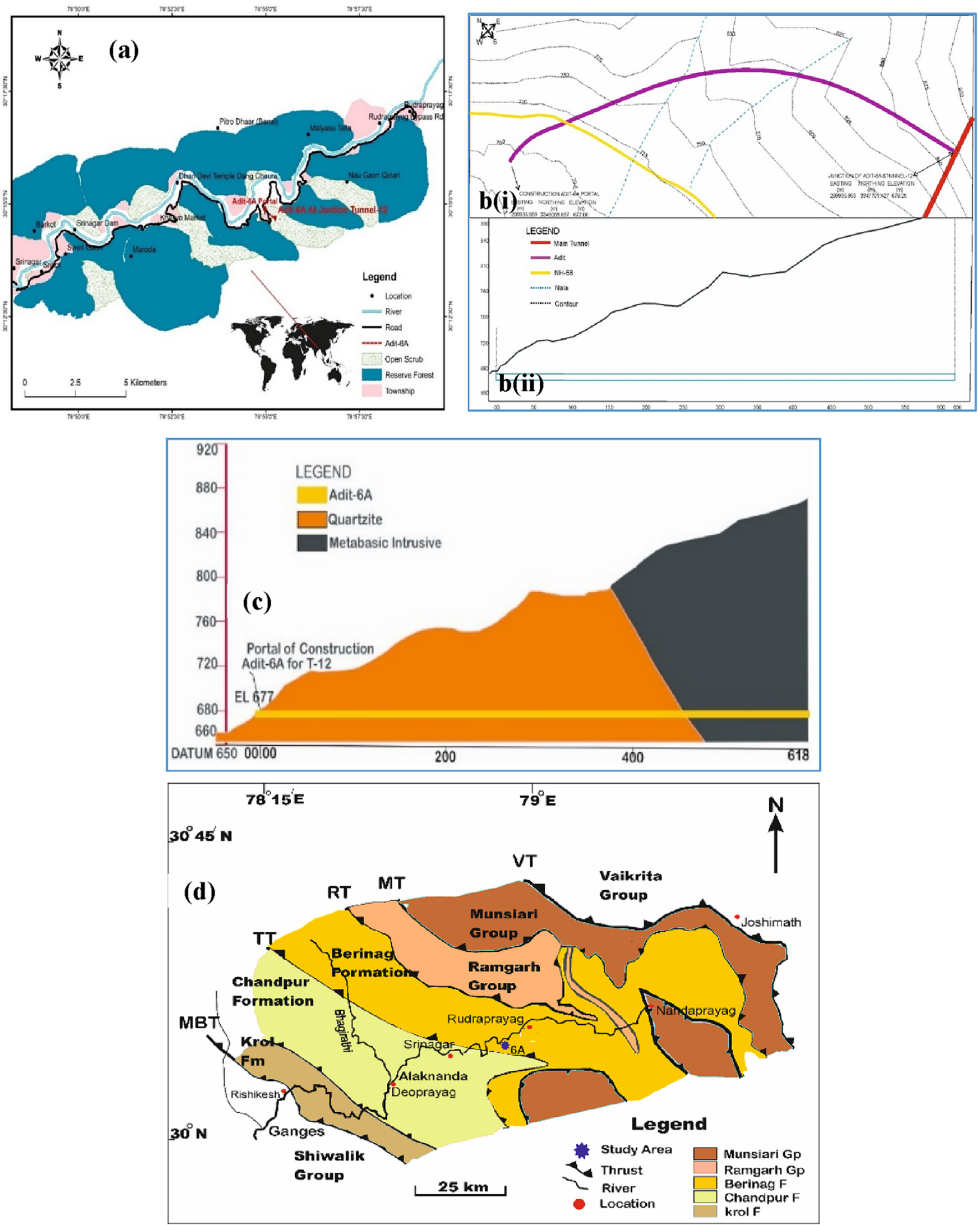
(**a**) Location of the study area, (**b**) layout map of the area (i) general topographic details, (ii) elevation profile along the tunnel, (**c**) geological section of the adit-6A, (**d**) simplified geological map of the study area showing numerous Himalayan litho-tectonic units, after Ahmad et al.^36^, *VT* Vaikrita thrust, *MT* Munsiari thrust, *RT* Ramgarh thrust, *TT* tons thrust, *MBT* main boundary thrust, *Gp* group, *Fm *formation.

The study area is located at Khankra, 13 km from Rudraprayag city in the S-W direction stretching from Karnaprayag to Rishikesh in Uttarakhand (Fig. 2a). The project is one of India’s most challenging railway tunnelling exercises. A horseshoe-shaped tunnel (adit) of 8.6 m finished diameter, called Tunnel-12 that was being constructed in the said area, was investigated for rockmass characteristics over a length of 618 m (Fig. 2b, c). The adit passes through quartzite and meta-basic intrusive rocks of the Garhwal Himalaya. The adit portal was excavated on the left bank of the Alaknanda River.

## Geology of the area

The topography of the area is dominated by lofty mountains with deep and wide valleys and high steep walls. The area is characterized by several landslides affecting the most superficial and altered portion of the bedrock. The landslides are activated generally during the monsoon.

The geology of the area has widely been investigated by a number of researchers over a wide span of time that include, e.g.,^37–43^. The Garhwal Group, which consists of the Meso-Proterozoic rocks, is exposed from Srinagar to Karnaprayag and is over-thrust by the North Almora Thrust over the Jaunsar Group. The Agastmuni, the Rautgara, the Pithoragarh, and the Berinag Formations make up the Garhwal Group (Fig. 2d).

The comprehensive geological map of the area after Ahmad^36^ is given in Fig. 2.

**Figure 3:**
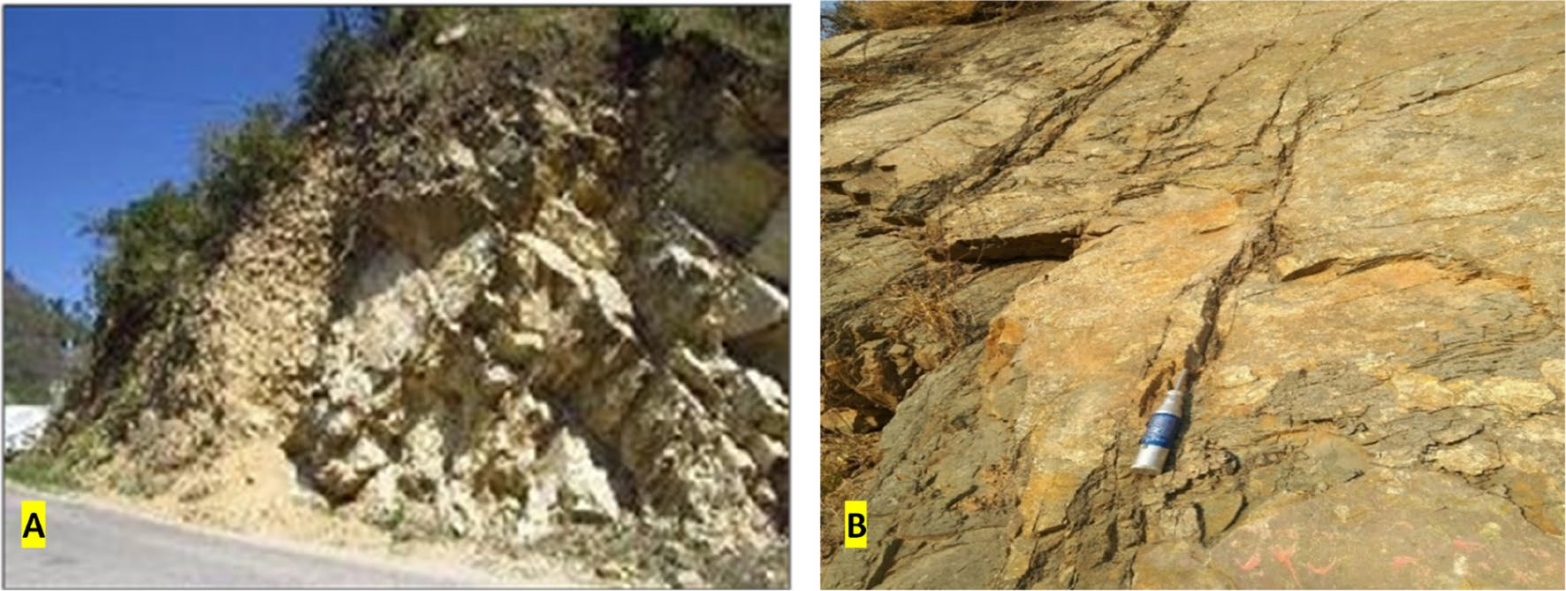
Exposure of the rock outcrop in the study area; (**A**) Berinag quartzite outcrop exposure, (**B**) massive quartz arenites outcropping with a moderate fracturing.

A geological cross-section is provided in Fig. 2c, wherein, the adit passes through two major lithologies viz. quartzite and metabasic rocks. The adit is driven in the quartzite of Berinag Formation of the Garhwal Group that mainly presents quartzite with subordinate purple phyllite and basic metavolcanic along with occasional chloritic, graphitic, and carbonaceous quartzite and metabasic intrusive rocks (Fig. 3A, B).

## Methodology

The International Society for Rock Mechanics (ISRM) criteria were adopted for recording of the rockmass variables during the field studies. Variables such as joint orientation, joint set number, aperture, roughness, spacing, filling types, persistence, and groundwater conditions were all measured during the investigations. A comprehensive mapping of the rockmass was carried out throughout the length of the adit. Observations of the discontinuities from the outcrops were used to determine the major joint sets along the alignment of the adit. The field observations were augmented with borehole data and other observations of relevant structural features.

The rocks in this area are slightly to moderately weathered. The wall surfaces of the joints are mostly rough, depending on the joint conditions. The aperture of the joints is tight to 1–5 mm filled with crushed and fine-grained materials like clay that is hard to soft in nature, respectively. In most parts of the adit, three sets of joints were observed. Quartzite is a white colour with fine to medium grain size showing rough or irregular planar surfaces that are moderately weathered with the intrusion of shear seams. The shear zone thickness is 10 to 15 cm with dry random joints. The uniaxial compressive strength of the quartzite, on an average, is 54 MPa with an RQD of 25% (average). The joints have 20 to 200 cm spacing and the surface of the joints is rough with a persistence of 1 to 5 m. The discontinuity orientation is fair to favourable with respect to direction of the adit.

## Engineering rockmass classification

As mentioned earlier, a variety of approaches are used to classify rockmass that are based on a group of variables^44,45^. However, in our case, different rockmass classification schemes like Rock Mass Rating (RMR)^7^, Rock Quality Index (Q), Rock Mass Number (Q_n_)^14^, Rock Mass Index (RMi)^2^, Geological Strength Index (GSI), Rock Structure Rating (RSR)^10^ and Rock Condition Rating (RCR), were worked out and evaluated for inter-correlation between the classifications.

The method of classifications along with the calculation used here are presented in Table 4. For the sake of simplicity only salient features of such classifications are provided as these are widely available in the published domain.

**Table 4:** Method for evaluation of the rockmass classifications in this study.

Sl. no.	Classification	Author(s)	General formula for calculation	Explanation/comments
1.	RMR	^7^	$RMR=UCS + RQD+ JS + JC + GW + JO$	Ratings for all 6 variables
2.	Q		$Q=\frac{RQD}{Jn}\times \frac{Jr}{Ja}\times \frac{Jw}{SRF}$	$\frac{RQD}{Jn}$ is overall structure of rock mass $\frac{Jr}{Ja}$ is Inter block shear strength $\frac{Jw}{SRF}$ is empirical factor describing the Active stress
3.	Q_n_	^14^	${Q}_{n}=\frac{RQD}{Jn}\times \frac{Jr}{Ja}\times Jw$	Modified Q
4.	RMi	^2^	$RMi={\sigma }_{cm} \times Jp$	
5.	GSI		$GSI=RMR-5 for GSI\ge 18 or RMR \ge 23$ $GSI=9{\text{ln}}Qn+44 for GSI <18$	
6.	RSR	^10^	$RSR=A+B+C$	Where, A is determined from the origin of rock type, B is determined by the discontinuities (average joint spacing, orientation of joints, and tunnelling direction) C is determined by the influence of ground water and joint conditions
7.	RCR	^72^	$RCR=RMR-Ratings (C \& JO)$	

*RQD* rock quality designation, *RMR* rock mass rating, *Q* tunnelling quality index, *RMi *rock mass index, *GSI* geological strength index, *RSR* rock structure rating, *RCR* rock condition rating, *Jn* number of joint sets, *Jr* joint roughness number, *Ja* joint alteration number, *Jw* water inflow, *SRF* stress reduction factor, ${\sigma }_{cm}$ uniaxial compressive strength of rock mass in MPa, *Jp* jointing parameter, *C* rating for crushing strength, *JO * adjustment for joint orientation.

The data include observations taken at and classification through nine methods, viz. RMi, Q, Qn, RMR, RMR_B_, RQD, RCR, RSR, and GSI. Data of joints were collected from the rock exposures along the alignment of the tunnel. The uniaxial compressive strengths of rocks were determined with the help of a Schmidt hammer from related charts, and the RQD was determined using the volumetric joint count. Some physical properties of the rocks encountered in the tunnel are given in Table 5.

**Table 5:** Basic rock properties obtained during the investigations in the study area.

Tunnel width	UCS	RQD avg	Water content	Joint alteration No	Joint roughness No	Joint orientation	Joint set No	SRF	Joint spacing	Persistence of joints	Weathering	Infilling	Structure
M	MPa	%	l/m			Degree			mm			mm	
8.5	60	16	10–12	2	1.5	J1: 160/45, J2: 220/25, J3: 140/75	3	2.5	60–200	Medium to low	Highly weathered	< 5	Blocky

Finally, from the data obtained the class of rocks were worked out using the methods given above and presented in Table 5. The complete data set of rock classifications of 34 locations along the alignment of the study is provided in Table 6.

**Table 6:** Values of the rockmass classification systems at the chainage on the adit-6A route.

Sl. no	Chainage	RMR	RMR_B_	Q	Qn	GSI	RMi	RQD	RSR	RCR
	00.00	40	42	0.33	0.832	35–40	0.75	17.5	40	15
	5.10	41	43	0.33	0.832	35–40	0.75	17.5	40	15
	6.5	41	43	0.33	0.832	35–40	0.75	20	40	15
	8.0	41	43	0.33	0.832	35–40	0.75	20	40	15
	9.2	40	45	0.33	0.832	35–40	0.75	20	40	15
	11.2	40	45	0.33	0.832	35–40	0.75	10	40	15
	13.2	40	45	0.33	0.832	35–40	0.75	10	42	15
	15.7	40	45	0.33	0.832	35–40	0.75	12.5	41	15
	18.5	40	45	0.33	0.832	35–40	0.75	12.5	40	15
	26.8	48	50	0.83	2.083	40–45	1.0	20	49	35
	39.5	48	50	0.83	2.083	40–45	1.0	20	49	35
	53.3	43	48	0.41	0.687	35–40	0.90	18	42	22
	103	38	40	0.27	0.687	35–40	0.75	12	39	12
	122	35	37	0.22	0.55	30–35	0.70	12	37	11
	123.6	35	37	0.22	0.55	30–35	0.70	12	37	11
	131.4	35	37	0.22	0.55	30–35	0.70	12	37	11
	132.5	35	37	0.22	0.55	30–35	0.70	12	35	11
	141.2	48	43	0.83	0.55	35–40	1.2	20	50	35
	142.60	36	38	0.22	0.55	35–40	0.70	12	35	11
	150.3	38	43	0.27	0.687	35–40	0.75	12.5	37	12
	153.2	38	43	0.27	0.687	35–40	0.75	12	38	12
	155.2	38	42	0.27	0.687	35–40	0.73	12	37	12
	167.2	45	47	0.55	1.375	40–45	0.85	19	39	24
	180.2	50	55	1.045	2.612	40–45	1.5	21	52	40
	185.2	50	52	0.99	2.695	40–45	1.2	20	50	39
	200.6	60	65	2.20	2.20	45–50	2.5	25	65	55
	222.3	48	53	0.88	2.20	35–40	0.95	20	50	38
	266.2	45	47	0.55	1.375	35–40	0.85	17	45	24
	288.9	49	51	0.91	2.291	40–45	1.0	19	48	39
	291	38	40	0.27	0.867	35–40	0.75	16	35	12
Shear zone	301.6 to 303.3	AGO	AGO	AGO	AGO	AGO	AGO	AGO	AGO	AGO
	307	49	54	0.93	2.343	35–40	1.3	19	50	40
	311	48	53	0.66	1.65	35–40	0.90	17	45	30
	322	38	43	0.27	0.687	30–35	0.75	12	37	12
	332.2	38	43	0.27	0.687	30–35	0.72	12	37	12

*RMR* rock mass rating, *RMR*_*B*_ basic rock mass rating, *Q* Q-system, *Qn* rock mass number, *RMi* rock mass index, *RQD* rock quality designation, *RSR* rock structure rating, *RCR* rock condition rating, *AGO* adverse geological occurrences like crushed or shear zones.

**Figure 4:**
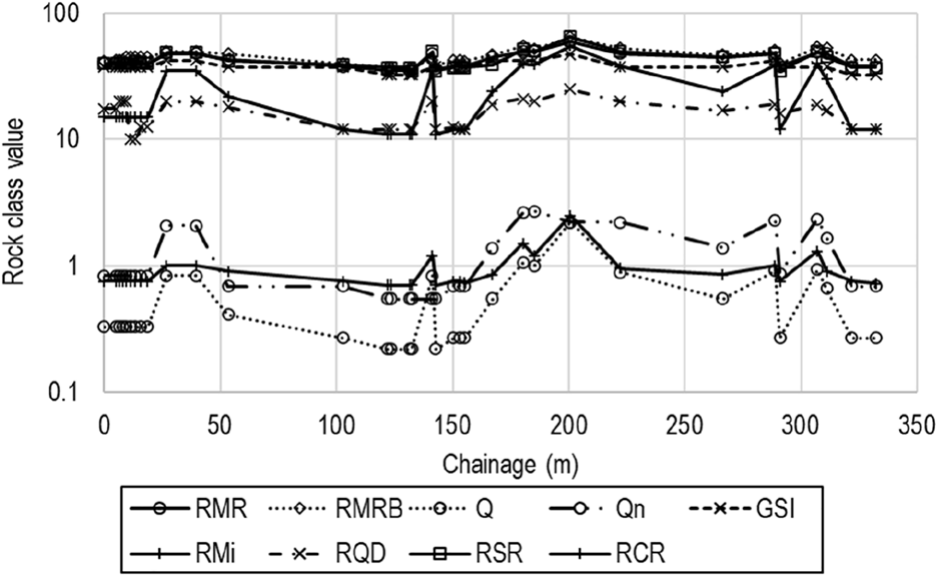
Values of different classifications worked for the adit along the chainage.

The values of the rock classifications worked out and presented in Table 6 are further plotted in Fig. 4.

It is evident from Fig. 4 that there is a significant variation of rockmass along the adit alignment. At chainage, 26.8 m to 39.5 m and 180.2 m to 200.6 m the white colour massive quartzite rock outcrops show higher values of all classifications, whereas the presence of shear zones in quartzite at chainage 122 m to 132.5 m show lowest values. To have a better overview of the data a descriptive statistic of values of rockmass classes obtained using different classifications are presented in Table 7.

**Table 7:** Descriptive statistic of values of rockmass from different classifications used in this study.

Classification	N	Range	Minimum	Maximum	Mean	Std. error	Std. dev	Variance	Skewness	kurtosis
RMR	34	25.00	35.00	60.0	42.24	1.00	5.82	0.90	0.79	0.90
RMR_B_	34	28.00	37.00	65.0	45.41	1.06	6.18	0.95	1.48	0.95
Q	34	1.98	0.22	2.2	0.52	0.07	0.40	2.46	8.19	2.46
Qn	34	2.15	0.55	2.7	1.16	0.12	0.70	1.02	-0.56	1.02
GSI	34	15.00	32.50	47.5	37.79	0.59	3.47	0.53	1.05	0.53
RMi	34	1.80	0.70	2.5	0.90	0.06	0.34	3.42	14.10	3.42
RSR	34	30.00	35.00	65.0	42.29	1.12	6.56	1.43	2.69	1.43
RCR	34	44.00	11.00	55.0	21.47	2.09	12.17	148.00	1.02	-0.06

The standard error and standard deviation are maximum for RCR with values of 2.09 and 12.17, respectively, followed by RSR, RMR and RMR_B_. Despite the errors, it is practically difficult to ascertain the discrepancies in the rock class values assigned by a classification system, as there is no measure or standard reference against which these can be calibrated. The only criterion we can follow for the comparison is the most used classification systems despite of their own disadvantages.

## Correlations between rock classes

**Figure 5:**
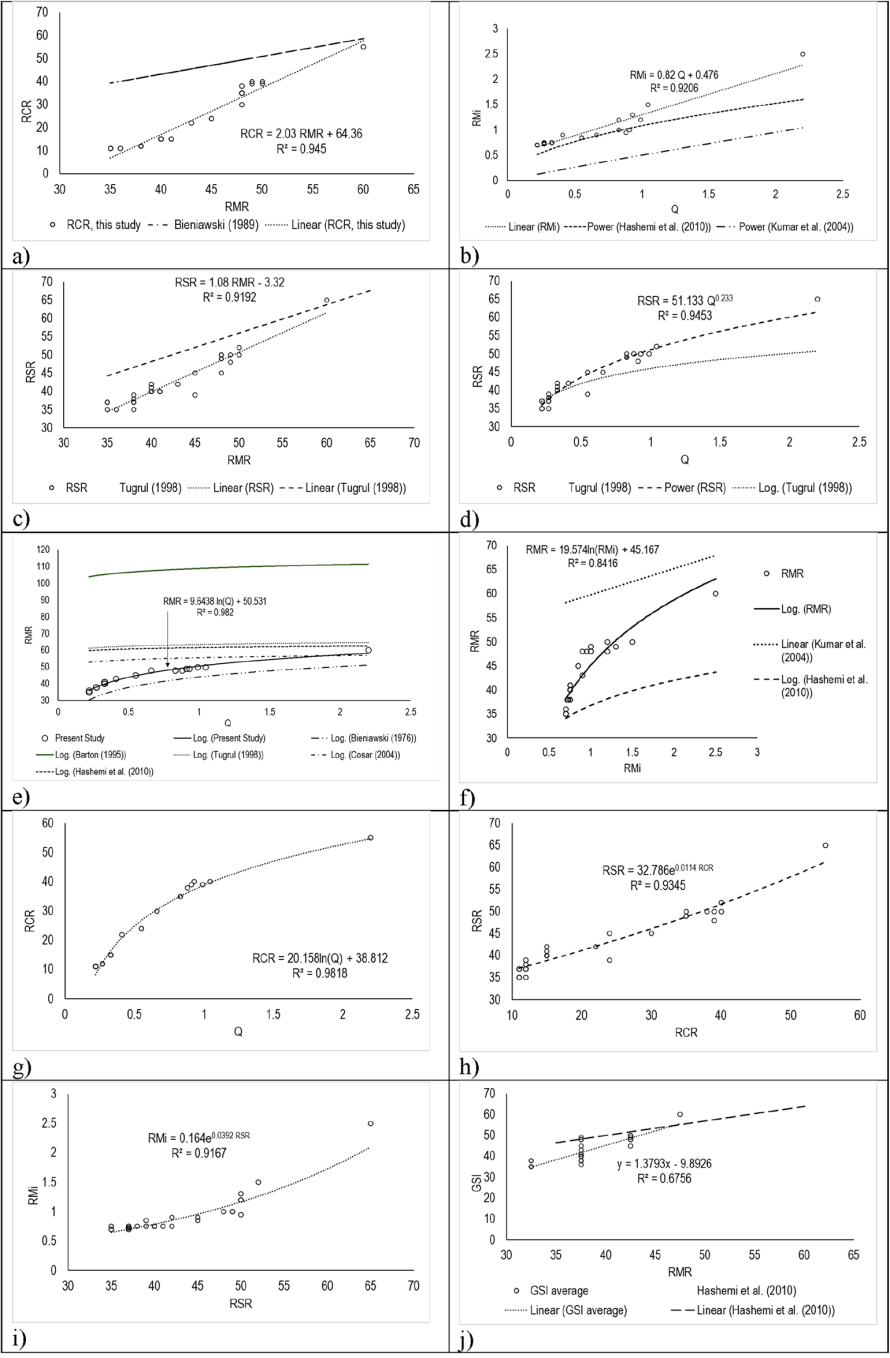
The best relationships observed in existing relationships (**a–i**) and new ones proposed, (**j**) is for the purpose of comparison only. Correlation relation between RMR and (**a**) Q-system, (**b**) RMi and (**c**) Qn. The additional correlations for RMR-Q (5 examples) and RMR-RMi (2 examples) were shown in "(**a**)" and "(**b**)" accordingly. Correlation between RMi and (**d**) RMR_B_, (**e**) Q-system, (**f**) RSR, (**g**) RCR, and (**h**) Qn. There are additional correlations for RMi-Q that may be compared, thus they are shown on the (**e**). (**i**) RMR, (**j**) Q, (**k**) RCR, and (**l**) Qn have all been correlated to RSR. The additional correlations for RSR-RMR^46^ are shown in '(**i**)' and RSR-Q are shown in '(**j**)'. Correlation between RCR and (**m**) RMR, (**n**) Q-system, and (**o**) Qn. Aside from the correlations for RCR-RMR^7^ and RSR-Q^14,47^, and (Hashemi et al., 2009), additional correlations for "(**m**)" and "(**o**)" are also included. Correlation between (**p**) GSI and RMR_B_, (**q**) RMR, (**r**) Q-system, (**s**) RMi, (**t**) RSR, (**u**) RCR and (**v**) Qn. We also include the other GSI-RMR correlation (Hashemi et al., 2004) in "(**q**)" for the sake of comparison.

In line with the objective of the study, the correlations between different rockmass classes worked out for the adit data acquired with the help of standard regression methods. As seen in Fig. 5a–i, the best goodness-of-fit model for every pair was selected from among a variety of possible options, including linear, logarithmic, exponential, and power models. The graphs Fig. 5a–i show the best-fit lines of the various research available for comparison. Based on the analysis of the data acquired and the classifications correlations thereof, the following groups can be identified:

1. Correlations that are existing particularly for RMR and Q—a comparative analysis of such relationships have been attempted.
2. Correlations that do not exist—new correlations are suggested with the data set of the area of study under investigation.

The relationships between different classification systems were worked out based on the best correlations obtained from the correlation analysis provided in Table 8.

**Table 8:** Correlation matrix of different classification systems.

Classification	Correlations								
	RMR_B_	Q	Q_n_	GSI	RMi	RQD	RSR	RCR	RMR
RMR_B_	**1.00**	0.89	0.85	0.78	0.82	0.71	0.90	0.90	0.94
Q	0.89	**1.00**	0.79	0.77	0.96	0.74	0.96	0.94	0.94
Qn	0.85	0.79	**1.00**	0.73	0.65	0.68	0.81	0.89	0.85
GSI	0.78	0.77	0.73	**1.00**	0.70	0.70	0.75	0.76	0.82
RMi	0.82	0.96	0.65	0.70	**1.00**	0.67	0.90	0.84	0.85
RQD	0.71	0.74	0.68	0.70	0.67	**1.00**	0.76	0.79	0.82
RSR	0.90	0.96	0.81	0.75	0.90	0.76	**1.00**	0.96	0.96
RCR	0.90	0.94	0.89	0.76	0.84	0.79	0.96	**1.00**	0.97
RMR	0.94	0.94	0.85	0.82	0.85	0.82	0.96	0.97	**1.00**

*RMR*_*B*_ basic form of rock mass rating, *Q* is Q-system, *Q*_*n*_ rock mass number, *GSI* geological strength index, *RMi* rock mass index, *RQD* rock quality designation, *RSR* rock structure rating, *RCR* rock condition rating, *RMR* rock mass rating.

Best correlations obtained for the data obtained in this study are highlighted.

It can be observed from Table 8 that RMR_B_ correlates well with Q, RSR and RSR; Q with RSR, RCR and RMR; Q_n_ with RCR; RMi with RSR; RSR with RCR and RMR and RCR with RMR. The correlations for which correlation exist and the new ones proposed are further explained with the help of Fig. 5a–i.

The equations thus developed are presented in Table 9 and have been arranged in decreasing order of adjusted R^2^ values. In addition, many other correlations (Table 9) were also attempted but these did not yield good results. However, these have been retained in Table 9 the for the benefit of the reader. The equations can be seen to behave in unique way for the rock type studied here.

**Table 9:** The relationship between different classification systems for the observation area.

Sl. no	Relationship	R^2^	Adjusted R^2^	MSE	RMSE	Comments
1.	*RCR* = *2.03 RMR* + *64.36*	0.95	0.94	155.36	11.39	New relationships
2.	*RMi* = *0.82 Q* + *0.476*	0.92	0.92	0.18	0.16	
3.	*RSR* = *1.08 RMR* + *3.32*	0.92	0.92	53.12	1.84	
4.	*RSR* = *51.13 Q*^*0.233*^	0.95	0.92	53.12	1.52	
5.	*RSR* = *32.786e*^*0.0114 RCR*^	0.90	0.92	53.12	2.2	
6.	*RCR* = *20.16 ln(Q)* + *38.812*	0.98	0.89	155.36	1.62	
7.	*RMR* = *9.64 ln(Q)* + *50.531*	0.98	0.88	34.18	0.77	
8.	*RMi* = *0.0013 RSR*^*1.7436*^	0.86	0.8	0.18	0.14	
9.	*RCR* = *19.6 ln(Qn)* + *21.585*	0.75	0.78	155.36	5.99	Correlations insignificant
10.	*RMR* = *19.57 ln(RMi)* + *45.16*	0.84	0.71	34.18	3.02	
11.	*RMR* = *9.3 ln(Qn)* + *42.29*	0.74	0.71	34.18	2.93	
12.	*RMi* = *0.552 e*^*0.0207RCR*^	0.80	0.7	0.18	0.16	
13.	*GSI* = *0.49 RMR* + *17.108*	0.68	0.67	9.85	1.95	
14.	*RMi* = *0.15 e*^*0.0383RMR*^_*B*_	0.80	0.66	0.18	0.17	
15.	*RSR* = *41.9 Qn*^*0.2166*^	0.64	0.64	53.12	3.91	
16.	*GSI* = *4.893 RMR*_*B*_^*0.5359*^	0.60	0.59	9.85	2.14	
17.	*GSI* = *4.64 ln(Q)* + *41.78*	0.64	0.59	9.85	2.06	
18.	*GSI* = *25.51 RCR*^*0.1328*^	0.57	0.56	9.85	2.23	
19.	*GSI* = *17.96 ln(RSR)—29.26*	0.55	0.55	9.85	2.28	
20.	*GSI* = *37.67 Qn*^*0.1266*^	0.57	0.51	9.85	2.25	
21.	*GSI* = *9.37 ln(RMi)* + *39.197*	0.54	0.47	9.85	2.31	
22.	*RMi* = *0.613 e*^*0.2936Qn*^	0.43	0.41	0.18	0.26	

The most significant relationship in our case was between RCR and RMR. However, in comparison to the relationship of Bieniawski^7^, there is a significant departure in the lower values with a good match at higher values (Fig. 5a). The case is reverse in the correlation between RMi and Q (Fig. 5b) where there is a departure towards the higher values in case of the relation provided by other authors^47,48^.

The relation between RSR and RMR (Fig. 5c) is almost similar to nature to the correlation between RCR and RMR (Fig. 5a). However in case of the former, the difference in prediction by the method of Tuǧrul^46^ is much less. The trends of our case and that of Tuǧrul^46^ are almost similar except that the departure is more in the lower values and minimum at higher values. The correlation between RSR and Q (Fig. 5d) presents a logrithmic nature as Q is a log function but there is difference in predictions by Tuǧrul^46^ at higher values of Q, the trend of which is practically assymptotic after a Q value of 1.

There are multiple relationships propsed for RMR and Q by various authors like^47–49^. However the correlation provided by^5^ is by far the best one that fits our data with a mild departure from the predicted values in its intercept (Fig. 5e). Other correlations (Fig. 5e) are practically behaving erratically possibly because of local customizations or errors in data representations. This points to the fact that the equation of Bienawski^50^ is an excellent presentation for evaluation of RMR from Q and can be used with good degree of confidence with local adjustments for variables as the trend is perfectly following the trend of the data developed for the data in discussion.

Another relation of RMR with RMi is of interest (Fig. 5f) as the data generated here shows a definite trend but the trends evaluated with the equations of provided by Kumar et al.^47^, that is a linear one, and by Hashemi^48^, behave erratically. The trend of Kumar et al.^47^ overpredicts and that of Hashemi^48^ underpredicts the values of RMi, if our data is used.

Few new equations, introduced here, that have not been observed earlier are the those of RCR with Q, RSR with RCR, RMi with RSR and RCR with Q. The trend of RCR presents an excellent correlation with Q (Fig. 5g) with a perfect logarithmic trend over the values of Q observed. Even higher values of the Q match perfectly with the values of RSR. The trend in case of RSR vs. RCR is of different nature as it presents an exponential relationship (Fig. 5h) as is also the case with the trend in case of RMi vs. RSR (Fig. 5i). There is a mild departure of these trendlines towards the higher values as there are less values in that region. This means that the relationships will be good for the values of RCR and RSR defined in the (Fig. 5h, i) or will need validation for the values beyond the said regions.

## Discussion

Rockmass classifications for engineering applications have witnessed a significant amount of research as documented through literature, application, and case studies. Further, the inter-correlations attempting by umpteen number of authors, documented in this paper, is a testimony to the fact.

It is important to mention that the correlations between rockmass classification systems are usually specific to a particular site and constraints generalisation. Also, that all classification systems use ratings for different measurable and non-measurable variables of the rockmass that are defined over a range of values to finally ascertain the class of the rock in a particular rock engineering project or application. The ratings are a major source of error in such classifications as these are based on the judgement of an individual and has been brought out by several authors, e.g.,^8,51^. Thus, the assignment is believed to be a function of cognition of a person taking the measurements^52^.

Also, there is considerable uncertainty over the accuracy of the ratings for certain variables. Also, researchers have investigated the convertibility of one classification to another to adhere to the specific requirements of the projects and in this process multitude of equations have emerged. The correlations in such cases may be one of the leading errors of estimation as perfect fit is not available in such conversions. This has resulted in variations in rock classification. Finally, different classifications have a different philosophy and as such different set of variables and ratings. On conversion to another system of rockmass classification, there is every chance of error. It will not be out of place to mention that there is a multiplication of errors. These variations thus produce poor results when attempting to identify correlations between any two rockmass classifications.

As demonstrated here, most of the inter-correlations between rockmass do not present good correlations when tested with our data. In addition to the what has been stated earlier, the reasons for the weak correlations between different classifications is that various systems evaluate parameters differently. This is the reasons some authors have developed fresh correlations while modifying the original classifications e.g., RCR and Qn are improved versions of earlier classifications systems. To improve correlations, e.g., Qn is derived from the Q-system with SRF set to 1 and RCR from RMR without UCS and joint orientation^14^. For higher groundwater conditions, the RMR'89 results show a strong correlation with GSI values when joint adjustment rates are ignored^7,53^. Such examples highlight the critical relevance of understanding the characteristics that affect the degree of correlation between classification systems.

However, despite of the said odds, one cannot preclude the conversions as at the end all the classifications represent the rockmass being excavated or supported. It is thus evident that for universal correlations between different systems, substantial data in different conditions is required. This work is thus an augmentation of the earlier data with fresh data from the Himalayan tunnelling scenario. While providing objective evaluation of the earlier correlations, some fresh correlations were developed for similar conditions.

Accordingly, the coefficients of determination viz. R^2^, and adjusted R^2^, mean-square error (MSE), and root-mean-square error (RMSE) of correlation between the classification systems have been evaluated in the present case (Table 8) to determine the stronger and weaker correlations between different classification systems. The weakest correlation coefficients were found between RMi, GSI, and Qn; between GSI and the other relations, such as GSI-RMR_B_, GSI-RMi, GSI-RSR, GSI-RCR, and GSI-Qn (Table 8). It may be pointed out that RMR and Q are calculated with the joint orientation parameter from GSI, but the Qn, RCR, RSR, RMi, and RMR_B_ relationships are not affected by it. The addition of the joint orientation in both the GSI and RMR classifications leads to a relatively high R^2^ between the two, as shown in (Fig. 5j).

Using correlations between classifications that do not compute values using joint orientation, we may assess the impact of other factors on the correlation coefficient. Therefore, we only consider parameter incompatibility in case of lesser correlation coefficients important, if it is consistent with classifications yielding greater correlation coefficients.

There is an incompatibility between groundwater conditions and the RMi-Qn and GSI-RMi, GSI-Qn, and GSI-RCR parameters with the lowest correlation coefficients, as well as the number of joint sets. However, the strong GSI-RMR_B_ relationship does not include groundwater conditions, so it is not considered an important parameter. Thus, it can be concluded that the number of joint sets is an important factor as lack of it leads to weak correlation coefficients. When looking for correlations between two different classifications, it is thus necessary to take into account the number of joint sets and the orientation of joint sets in both the classification systems under comparison.

## Conclusions

Inter-correlation relationships between different rockmass classifications are proposed here based on field and laboratory data collected along an adit alignment of a tunnel project in the Himalayan region. The rocks investigated belong to a quartzite rock and present a significant range of rock class values in different classification systems. The inter-correlation between a host of classification worked out for our data indicated that many of these do not present good convertibility when evaluated over adjusted R^2^ values. The best correlations that emerged from the analysis are between RCR-RMR, RMi-Q, RSR-RMR, RSR-Q, RMR-Q, RMR-RMi, RCR-Q, RSR-RCR, RMi-RSR wherein the correlation between RMR and Q is seen to behave in an excellent manner. A comparative analysis with other published cases indicated that there is a significant deviation in the correlation with those developed in this paper. New correlations in addition to the above include that between RCR & Q, RSR & RCR, RMi & RSR out of which RCR-Q presents the best fit. With the addition of JS and JO in some classifications, it is seen that the correlations with other classifications improve and hence it is imperative that JS and JO should invariably form part of the classification. Also, this study, though limited to a particular type of rock in Himalayan region, enhances the database and know-how of classifications in such formations and can be valuable for Rock Engineers and Geologists, equally. Additional data on different rock types and geological conditions, new and reliable measurement methods that have better repeatability with enhance the understanding of the subject. In addition, a considerable database in Himalayan conditions is desired that can well be put to deep learning techniques and correlated with the ground response.

## Data Availability

Data can be made available by the corresponding author on request.
